# The Inherited Intestinal Microbiota from Myeloid-Specific ZIP8KO Mice Impairs Pulmonary Host Defense against Pneumococcal Pneumonia

**DOI:** 10.3390/pathogens12050639

**Published:** 2023-04-25

**Authors:** Derrick R. Samuelson, Deandra R. Smith, Kelly C. Cunningham, Sabah Haq, Daniel N. Villageliú, Christi M. Ellis, Niaz Bahar Chowdhury, Amanda E. Ramer-Tait, Jeffrey D. Price, Daren L. Knoell

**Affiliations:** 1 Department of Internal Medicine-Pulmonary Division, College of Medicine, University of Nebraska Medical Center, Omaha, NE 68198-5910, USA; 2 Nebraska Food for Health Center, University of Nebraska-Lincoln, Lincoln, NE 68508, USA; 3 Department of Pharmacy Practice and Science, College of Pharmacy, University of Nebraska Medical Center, Omaha, NE 68198-6120, USA; 4 Department of Chemical and Biomolecular Engineering, University of Nebraska-Lincoln, Lincoln, NE 68588-0643, USA; 5 Department of Food Science and Technology, University of Nebraska-Lincoln, Lincoln, NE 68588-6205, USA

**Keywords:** zinc transporter, microbiome, gut-lung axis, pneumonia, host defense

## Abstract

Intestinal dysbiosis increases susceptibility to infection through the alteration of metabolic profiles, which increases morbidity. Zinc (Zn) homeostasis in mammals is tightly regulated by 24 Zn transporters. ZIP8 is unique in that it is required by myeloid cells to maintain proper host defense against bacterial pneumonia. In addition, a frequently occurring ZIP8 defective variant (*SLC39A8* rs13107325) is strongly associated with inflammation-based disorders and bacterial infection. In this study, we developed a novel model to study the effects of ZIP8-mediated intestinal dysbiosis on pulmonary host defense independent of the genetic effects. Cecal microbial communities from a myeloid-specific *Zip8* knockout mouse model were transplanted into germ-free mice. Conventionalized ZIP8KO-microbiota mice were then bred to produce F1 and F2 generations of ZIP8KO-microbiota mice. F1 ZIP8KO-microbiota mice were also infected with *S. pneumoniae,* and pulmonary host defense was assessed. Strikingly, the instillation of pneumococcus into the lung of F1 ZIP8KO-microbiota mice resulted in a significant increase in weight loss, inflammation, and mortality when compared to F1 wild-type (WT)-microbiota recipients. Similar defects in pulmonary host defense were observed in both genders, although consistently greater in females. From these results, we conclude that myeloid Zn homeostasis is not only critical for myeloid function but also plays a significant role in the maintenance and control of gut microbiota composition. Further, these data demonstrate that the intestinal microbiota, independent of host genetics, play a critical role in governing host defense in the lung against infection. Finally, these data strongly support future microbiome-based interventional studies, given the high incidence of zinc deficiency and the rs13107325 allele in humans.

## 1. Introduction

The influence of the gut microbiota on lung immunity, referred to as the gut–lung axis, is vital for protection against invading pathogens. The human body is colonized by a vast number of microbes, with the gut being the most densely colonized organ. Immune homeostasis is dependent on a microbiome that provides microbial metabolites for appropriate maturation and maintenance of the immune system [1]. In humans, environmental factors, including dietary intake of key nutrients, can shift the gut microbiota by decreasing the abundance of beneficial bacterial species while promoting the outgrowth of pathogenic species. Gut microbial imbalances linked to a disease state, referred to as dysbiosis, have become a topic of interest in the development and pathogenesis of many different diseases. Importantly, intestinal dysbiosis negatively impacts the regulation of the gut–lung axis and is linked to increased susceptibility to respiratory infections [2].

Community-acquired pneumonia (CAP) is a leading cause of morbidity and mortality worldwide. *Streptococcus pneumoniae* (pneumococcus) remains the most commonly identified cause of CAP in the U.S. [3,4,5,6]. The incidence of CAP continues to rise, contributing to increased hospitalizations and mortality [7,8]. A major cause of CAP is due to compromised immune function [9]. Zinc (Zn) is required for proper immune function [10,11,12], and insufficient dietary intake is highly prevalent within vulnerable populations [13,14]. Zn-deficient subjects are more susceptible to bacterial and viral pathogens [15] and have a higher incidence of pneumonia [16,17,18], while Zn supplementation can reduce the incidence of pneumonia [19,20,21].

Zn homeostasis in mammals is tightly regulated by a constellation of 24 Zn transporters. Our group was the first to reveal that the Zn transporter, ZIP8, is unique in that it is required by myeloid-lineage cells to maintain proper host defense against bacterial pneumonia [22,23,24,25]. Recent human studies have revealed that a frequently occurring ZIP8 variant dysfunctional allele (rs13107325; Ala391Thr risk allele) is strongly associated with inflammation-based disorders [26,27] and bacterial infection [28]. Based on this, our laboratory created a novel myeloid-specific *Slc39a8(-/-)* knockout (ZIP8KO) mouse model and determined whether ZIP8 loss altered host protection against pneumococcal pneumonia via changes in the gut microbiota. Studies were conducted primarily in *S. pneumoniae*-infected F1 progeny, who were the offspring of germ-free parent mice subjected previously to cecal microbiota transplant. Comparison of the microbiota of fecal samples between WT and ZIP8KO mice revealed substantial differences in microbial composition, as well as colon cellular morphology. Most striking, following the instillation of pneumococcus into the lung, we observed a significant increase in weight loss, inflammation, and morbidity in mice that harbored microbiota from ZIP8KO mice when compared to WT (control) counterparts. These results revealed a novel and potential vital axis that exists between Zn and ZIP8-mediated alteration of the gut microbiome and host defense against pneumococcus (and likely other pathogens). Based on these novel observations, we established that Zn dyshomeostasis causes significant alterations to the gut microbiome, which adversely impacts immune function in the lung in response to bacterial infection.

## 2. Materials and Methods

### 2.1. Animal Studies

All animals were maintained under specific pathogen-free conditions in the Animal Resource Facility at the University of Nebraska Medical Center. Food and water were provided ad libitum. The research protocol used in these studies was approved by the Institutional Animal Care and Use Committee of the University of Nebraska Medical Center. All methods involving animal care and procedures in this research protocol were performed in accordance with the NIH and Office of Laboratory Animal Welfare (OLAW) guidelines. Conditional *Slc39a8* knockout mice, referred to as ZIP8KO, were generated as previously described [22,25]. C57BL/6J wild-type counterparts were purchased from Jackson Labs and bred for experimental procedures.

### 2.2. Microbiota Collection and Cecal Microbial Engraftment

Cecal microbiota inoculum was prepared under anaerobic conditions. Specifically, frozen cecal content was homogenized in sterile 10% glycerol PBS and passed through a 330 μm filter to remove large particulate matter and facilitate gavage. C57BL/6 germ-free mice from the Nebraska Gnotobiotic Mouse Program were transferred from gnotobiotic isolators to a positive-pressure individually ventilated cage rack system and orally gavaged with 100 μL of ZIP8KO or WT cecal microbiota. Mice were then maintained in sterile caging on autoclaved bedding and fed sterilized chow and water for the remainder of the study. ZIP8KO or WT conventionalized mice were bred using standard laboratory practices. F1 and F2 generations of ZIP8KO- or WT-microbiota colonized mice were then used to assess all experimental endpoints. Twelve- to thirteen-week-old F1 and F2 offspring were infected with *S. pneumoniae* or PBS via oropharyngeal aspiration and sacrificed at indicated time points post-infection (see below).

### 2.3. DNA Sequencing of the 16s rRNA Gene

16s rRNA sequencing was performed in the Genomics Core at the University of Nebraska Medical Center. Briefly, the QIAamp PowerFecal ProDNAKit was used to extract genomic DNA from the flash-frozen cecal contents (Qiagen Valencia, CA, USA). The V3/V4 region of the 16S rRNA gene was then amplified for each sample, starting with 12.5 ng of DNA. The 16S Amplicon PCR Forward Primer was 5-TCGTCGGCAGCGTCAGATGTGTATAAGAGACA GCCTACGGGNGGCWGCAG-3, while the 16S Amplicon PCR Reverse Primer was 5-GTCTCGTGGGCTCGGAGATGTGTATAAGAGACAGGACTACHVGGGTATCTAATCC-3. Using the Nextera XT Index kit (Illumina FC-131-1001), dual indices and Illumina sequencing adapters were inserted after the amplicons were generated. On an Illumina MiSeq device employing V3 chemistry, 300 bp paired-end sequencing of the resultant libraries was performed. Internal sequencing controls (a positive mock community control and a negative water control) were also analyzed.

### 2.4. Sequence Analysis

Raw sequence data were processed using R and the R packages: DADA2 v1.1.5; Phyloseq v1.16.2; DESeq2 v1.20.0; and vegan v2.3-5 [29,30,31,32,33,34]. Sequences were truncated, denoised, chimera-filtered, and clustered into sequence variants using DADA2. Operational taxonomic units (OTU) were generated in DADA2 by taxonomic classification of sequence variants using the SILVA reference database v132. Alpha diversity (Chao1 index), beta-diversity, taxonomic summaries, and differentially abundant ASVs were determined using microbiome analyst [35].

### 2.5. Culture, Quantification, and Instillation of Streptococcus pneumoniae

The *S. pneumoniae* strain JWV500 (D39hlpA-gfp-Cam’), a generous gift from Dr. Jan-Willem Veening (University of Lausanne, Switzerland), was grown to mid-log phase, aliquoted, frozen, and stored at –80 °C. For lung infection studies, bacteria were grown to log phase in Remel Mueller Hinton Broth (ThermoFisher Scientific, Waltham, MA, USA) supplemented with 32 mg/mL chloramphenicol. For quantification of pneumococci, serial dilutions of the bacteria were plated on Remel blood agar plates (ThermoFisher Scientific) and incubated at 37 °C with 5% CO_2_ overnight to determine colony-forming units (CFUs). For oropharyngeal aspiration, mice were lightly anesthetized using 2% isoflurane and 1 L/min of oxygen and instilled with 4 × 10^8^ CFU of *S. pneumoniae* in 100 µL of phosphate-buffered saline (PBS). The dose of *S. pneumoniae* was confirmed by serial dilutions.

### 2.6. S. pneumoniae Lung and Spleen Quantification

The *S. pneumoniae* burden in the lungs and spleens of mice was measured by real-time quantitative PCR of the *lytA* gene, using a TaqMan Fast Advanced Master Mix (Sigma-Aldrich, St. Louis, MO, USA), according to manufactures specifications. The following primer sequences were used: LytA forward, 5’-ACGCAATCTAGCAGATGAAGCA-3’ and LytA Reverse, 5’-TCGTGCGTTTTAATTCCAGCT-3’. The probe (5’-TGCCGAAACGCTTGATACAGGGAG-3’) was labeled at the 5′ end with 6-carboxyfluorescein (FAM; IDT, Coraville, IA, USA), an internal ZEN Quencher, and on the 3′ end with the Iowa Black FQ Quencher (3IABkFQ; IDT, Coraville, IA, USA). The assays were carried out in a final 20 μL reaction volume, with 2.0 μL of sample DNA. The primers and probe were added at 200 nM concentrations. A no-template control and an *S. pneumoniae*-positive DNA control were included in every run. DNA was amplified with the 7500 Real-Time PCR system (Applied Biosystems, Waltham, MA, USA) by using the following cycling parameters: 95 °C for 10 min, followed by 40 cycles of 95 °C for 15 s and 60 °C for 1 min. The pulmonary burden of *S. pneumoniae* (CFU/Lung) was calculated based on a standard curve of purified *S. pneumoniae* DNA derived from a known CFU value.

### 2.7. Bronchoalveolar Lavage (BAL) Fluid Analyses

Lungs were lavaged three times with 1 mL of ice-cold PBS. Total cell counts were determined using the Bio-Rad TC20 automated cell counter (Bio-Radd, Hercules, CA, USA), and differential cell counts were determined on cytospin-prepared slides stained with Hema-3 (ThermoFisher Scientific). Cytokine, chemokine, and total protein levels were measured using commercially available ELISA kits according to manufacturers’ instructions (BioLegend, R&D Systems, San Diego, CA, USA).

### 2.8. Lung and Colon Histology

Whole lungs were inflated with 10% formalin (ThermoFisher Scientific) to preserve pulmonary architecture. Mid-colon segments were fixed in 10% formalin. Lungs and colons were processed, paraffin-embedded, sectioned (4–5 µm), and stained with hematoxylin and eosin by the UNMC Tissue Sciences Core Facility. Slides were scanned using the Leica Aperio CS2 (Leica Biosystems, Deer Park, IL, USA), and images were acquired (20×) using the Leica ImageScope software. Colonic cross-sections were blindly scored using a previously published scoring system that assesses crypt architecture, cell infiltration, muscle wall thickening, and crypt abscess [36]. Fixed mid-colon segments were stained with periodic acid-Schiff (PAS) and alcian blue (AB) for the detection of intestinal goblet cells. The number of PAS/AB+ goblet cells/10 crypts was determined by counting the cells in four different areas in each section. Additionally, the percentage of PAS/AB+ and only AB+ cells was determined in each section by a blinded pathologist to semi-quantitate the differences in the two types of goblet cell staining.

### 2.9. Serum SP-D and FABP1 Quantification

Serum was collected at specified time sacrifice points using BD serum separator tubes (BD Biosciences, San Jose, CA, USA). Serum was then subject to SP-D and FABP1 ELISAs, according to the manufacturer’s specifications (R&D systems, Minneapolis, MN, USA).

### 2.10. Zn Quantification of Serum and Tissue

Solid tissues were dried, weighed, and digested in 1 mL mixed acid solution (nitric acid:perchloric acid [1:2]) at 80 °C for 4 to 6 h. Liquid samples were digested with nitric acid (1%) overnight. Samples were then subjected to atomic absorption spectroscopy (AAS) (AAnalyst 400; PerkinElmer, Waltham, MA, USA).

### 2.11. Statistics

Statistical analyses were performed using GraphPad Prism version 9.1 (GraphPad Software, La Jolla, CA, USA) and the R package vegan. Results are shown as the mean ± standard error of the mean. A *p* < 0.05 and a false discovery rate (FDR) *q*-value < 0.05 were deemed significant. Sample size and number of replicates are indicated in each respective figure legend. Statistical significance was assessed using Welch’s test for comparisons between two groups and a one-way analysis of variance (ANOVA) with Sidak’s multiple comparison test for comparisons among three or more groups. Survival curves were analyzed using Mandel–Cox modeling. Survival data at each day post-infection were assessed using the Chi-square test, and an expected 50% loss on each day starting 48 h post-infection for both groups. The statistical significance of the different microbiome measurements was assessed as follows: Alpha-diversity significance was inferred using ANOVA. Beta-diversity significance was inferred via permutational multivariate analysis of variance using distance matrices with corrections for multiple comparison via FDR. Differentially abundant ASVs were determined using linear discriminant analysis effect size (LefSe). A significance level of the FDR adjusted *p* < 0.05 and a linear discriminant analysis (LDA) score > 2 were used to determine the taxa that best characterize each phenotype.

## 3. Results

### 3.1. ZIP8-Mediated Dysbiosis Is Maintained in Germ-Free F0 Mice and F1 and F2 Offspring

**Figure 1:**
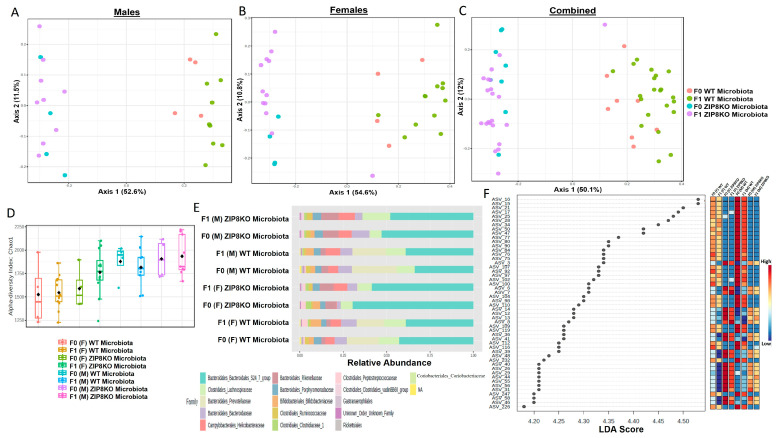
Microbiota differences between ZIP8KO and WT mice are maintained in the first generation of mice following colonization of germ-free breeders. Composition of cecal microbiota from F0 and F1 mice was analyzed by 16s rRNA sequencing. β-diversity demonstrated by PCoA of Bray–Curtis dissimilarity in (**A**) male F0 and F1 mice with WT and ZIP8KO microbiota, (**B**) female F0 and F1 mice with WT and ZIP8KO microbiota, and (**C**) both male and female F0 and F1 mice with WT and ZIP8KO microbiota. (**D**) α-diversity determined by Chao1 index. (**E**) Taxonomic comparison of microbiota. (**F**) Differentially abundant ASVs as determined by LefSe using the Kruskal–Wallis rank sum test, followed by Linear Discriminant analysis.

**Figure 2:**
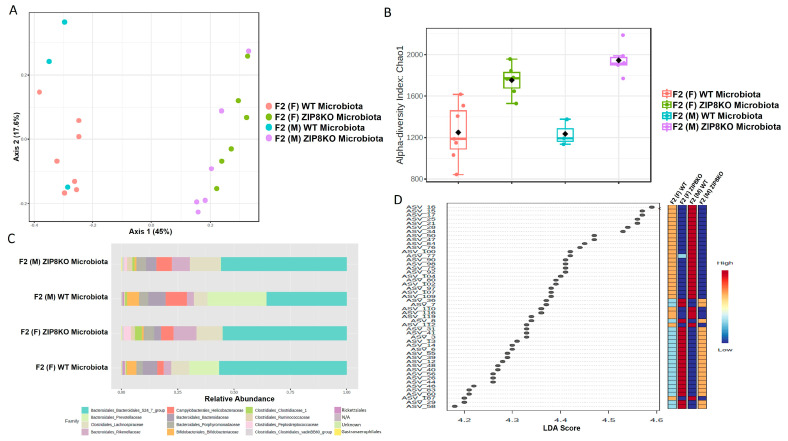
Microbiota differences are maintained in the second generation of colonized mice. Composition of cecal microbiota from F2 mice was analyzed by 16s rRNA sequencing. β-diversity demonstrated by PCoA of Bray–Curtis dissimilarity in (**A**) male and female F2 mice with WT and ZIP8KO microbiota, (**B**) α- diversity determined by the Chao1 index. (**C**) Taxonomic comparison of microbiota. (**D**) Differentially abundant ASVs as determined by LefSe using the Kruskal–Wallis rank sum test, followed by Linear Discriminant analysis.

Previously, we reported that ZIP8 loss in myeloid lineage cells altered the composition of the intestinal microbiota and that these changes contributed to impaired host defense against bacterial pneumonia [37]. However, given the limitations of antibiotic depletion and conventionalization methods, we sought to further define the role of ZIP8-mediated-dysbiosis on pulmonary host defense, independent of genetics. Specifically, cecal contents from either donor WT or ZIP8KO mice were transplanted into germ-free mice. Following microbial stabilization, the conventionalized F0 mice were bred to produce WT- or ZIP8KO-microbiota-associated F1 and F2 generation of mice. The microbiota was then evaluated in F0, F1, and F2 mice. F0, F1, and F2 mice from WT and ZIP8KO recipients separated into distinct clusters as shown by Bray–Curtis diversity and principle coordinate ordination (PCoA) (Figure 1A–C). b-diversity was significantly different between the WT and ZIP8KO microbiota across F0, F1, and F2 generations and maintained in both male and female mice (Figure 1A,B and Figure 2A). a-diversity was not significantly affected in the F0 and F1 generations except for F1 a-diversity between female WT and ZIP8KO microbiota (Figure 1D). In contrast, a-diversity was significantly different between F2 WT and ZIP8KO microbiota in both males and females (Figure 2B). Cecal microbiota composition was further analyzed using taxonomic summaries from amplicon sequence variants (ASVs). Taxonomic summaries revealed dissimilarity among F0, F1, and F2 WT and ZIP8KO microbiota (Figure 1E and Figure 2C). Interestingly, the genus Prevotellaceae_UCG_001 was reduced in F0, F1, and F2 ZIP8KO microbiota compared to WT microbiota (Figure 1F and Figure 2D), whereas the genus Bacteroidales_S24-7 was enriched in the F0, F1, and F2 ZIP8KO microbiota compared to WT microbiota (Figure 1F and Figure 2D).

### 3.2. Alteration of Gut Anatomical and Cellular Morphology

**Figure 3:**
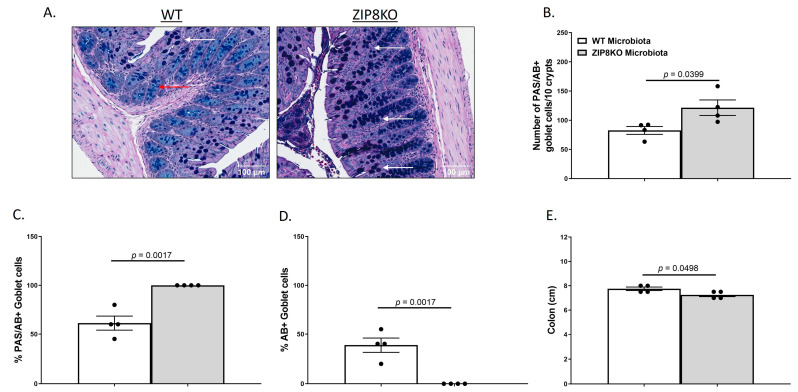
Myleoid-specific loss of Zip8 alters GI goblet cell numbers and composition. (**A**) Representative 20× images of PAS/AB-stained colonic cross-sections from male WT and ZIP8KO mice. (**B**) Number of PAS/AB+ goblet cells/10 crypts. (**C**,**D**) Quantification of the percentage of PAS/AB-stained areas (white arrows) and only AB-stained regions of each section (red arrows). (**E**) Colon length. Bars represent the mean ± SEM, and dots represent individual mice. *p*-values indicated in the figures were determined by Welch’s *t*-test.

To investigate the gut in WT and ZIP8KO donor mice, we compared the gross anatomy and cellular morphology of the colon. Histological examination of the colon of naïve male WT and ZIP8KO mice revealed no significant differences in intestinal inflammatory scores (Supplementary Figure S1). However, we observed an increased number of goblet cells in ZIP8KO mice. To further explore this finding, we stained colon sections with PAS/AB stain (Figure 3A). Consistent with our observation of H&E stained sections, the number (Figure 3B) and percentage (Figure 3C) of PAS/AB+ colonic goblet cells were significantly increased in male ZIP8KO mice compared to male WT counterparts. Further, the composition of mucin was significantly altered in ZIP8KO mice by a marked decrease in the acidic mucin-stained AB+ colonic goblet cells in the male ZIP8KO group compared to the WT group (Figure 3D). Finally, the colon length in the untreated male ZIP8KO mice was significantly decreased in males compared to their WT counterparts.

**Figure 4:**
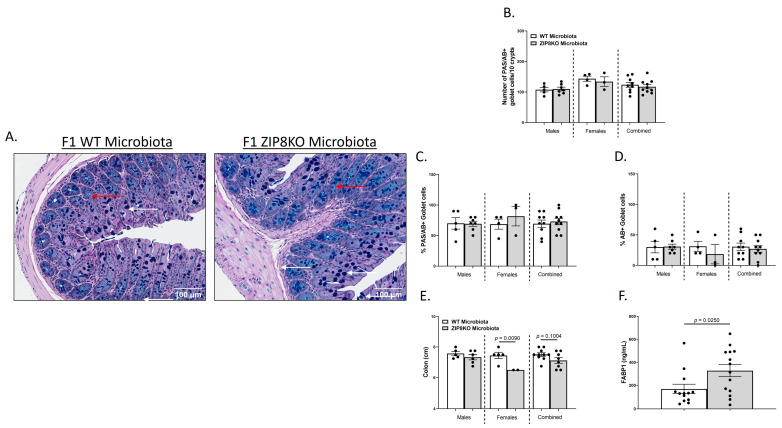
ZIP8-associated dysbiosis contributes to alterations in GI goblet cell numbers and composition. (**A**) Representative 20× images of PAS/AB-stained colonic cross-sections from male F1 WT and F1 ZIP8KO-microbiota mice. (**B**) Number of PAS/AB + goblet cells/10 crypts. (**C**,**D**) Quantification of the percentage of PAS/AB-stained areas (white arrows) and only AB areas in each section (red arrows). (**E**) Colon length. (**F**) Serum FABP1 measurement of combined genders. Bars represent the mean ± SEM, and dots represent individual mice. *p*-values are indicated in the figure and were determined by Welch’s *t*-test.

**Figure 5:**
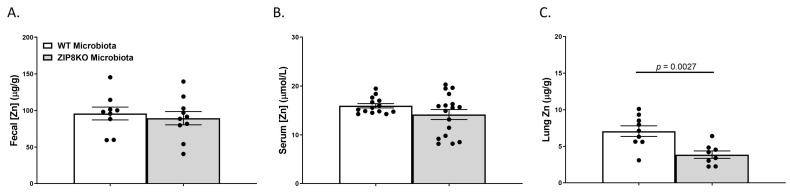
ZIP8-associated dysbiosis contributes to alterations in lung Zn levels. Zn levels in (**A**) feces, (**B**) serum, and (**C**) lung tissue. Bars represent the mean ± SEM, and dots represent individual mice. *p*-values are indicated in the figure and were determined by Welch’s *t*-test.

We also investigated the gross anatomy and cellular morphology of the colon of F1 ZIP8KO-microbiota mice. Similar to the donors, there were no significant differences in the histological inflammatory scores of the colon in the F1 WT and ZIP8KO-microbiota mice (Supplemental Figure S2). Likewise, there was no significant increase in colonic goblet cell number and mucin composition in the untreated F1 WT and ZIP8KO mice (Figure 4A–D). However, the number of PAS/AB+-or AB+-stained colonic goblet cells was not significantly different in the F1 ZIP8KO mice when compared to the WT mice in both males and females (Figure 4B,D). Colon length was significantly decreased in female F1 ZIP8KO and was shorter but not significant in F1 male mice (Figure 4E). F1 ZIP8KO-microbiota mice also exhibited marked increases in intestinal permeability, as measured by circulating levels of intestinal fatty-acid-binding protein (FABP1) (Figure 4F). Collectively, these data suggest that the microbiota from F1 ZIP8KO mice contributes to altered intestinal goblet cell numbers, as well as epithelial damage, but that host genetics play a larger role. Finally, we assessed local (fecal), circulating, and distal (lung) levels of Zn in the F1 ZIP8KO-microbiota mice. Local and circulating levels of Zn were not different between F1 WT and ZIP8KO-microbiota mice (Figure 5A,B). However, to our surprise, F1 ZIP8KO-microbiota mice exhibited reduced Zn lung tissue levels compared to F1 WT-microbiota mice (Figure 5C). These data suggest that the microbiota may also contribute to regulating Zn levels in distant organs.

### 3.3. ZIP8-Mediated Dysbiosis Increases Lung Leukocyte Recruitment and Inflammation

**Figure 6:**
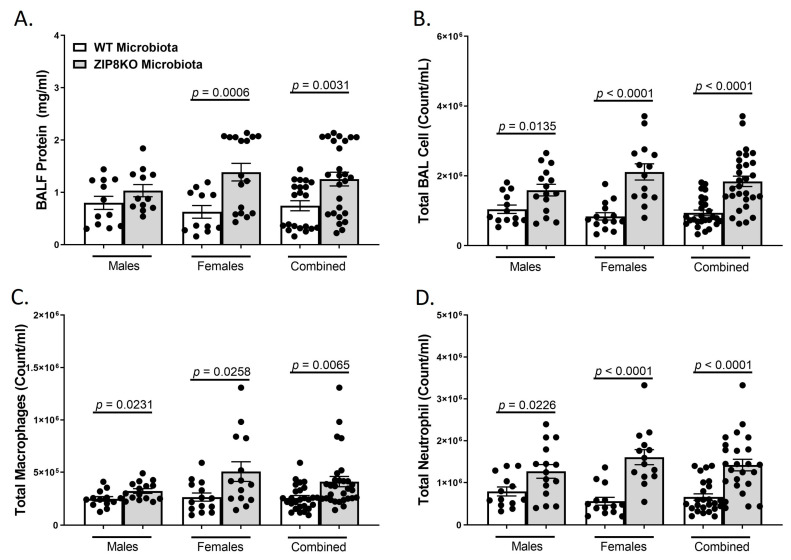
ZIP8-associated dysbiosis increases pulmonary immune cell numbers. F1-microbiota mice were infected with *S. pneumoniae*, and the number of BALF immune cells was assessed. (**A**) Protein levels from BALF. (**B**) Total BAL counts. (**C**) Total macrophages post-infection. (**D**) Total neutrophils post-infection. Bars represent the mean ± SEM, and dots represent individual mice. *p*-values are indicated in the figure and were determined by Welch’s *t*-test.

To determine the role of ZIP8-mediated intestinal dysbiosis on pulmonary host defense against pneumococcal infection, germ-free mice were colonized with the microbiota from WT and ZIP8KO mice. Male and female F1 progeny (age 12–13 weeks) were then administered 4 × 10^8^ CFU of *S. pneumoniae* in the lung via oropharyngeal aspiration and euthanized 48 h later. Analysis of bronchoalveolar lavage (BAL) fluid from the lungs revealed that bacterial instillation resulted in a significant increase in BAL protein, indicative of lung injury (Figure 6A). F1 ZIP8KO-microbiota mice also exhibited increased total numbers of leukocytes in their airways compared to WT recipient mice, with significantly more cells in both male and female F1 ZIP8KO recipients (Figure 6B). This corresponded with significant increases in macrophages (Figure 6C) and neutrophils (Figure 6D) and with an even greater accumulation of both cell types in females when compared to males (Figure 6B–D) There were no significant changes in lymphocytes across different groups. 

**Figure 7:**
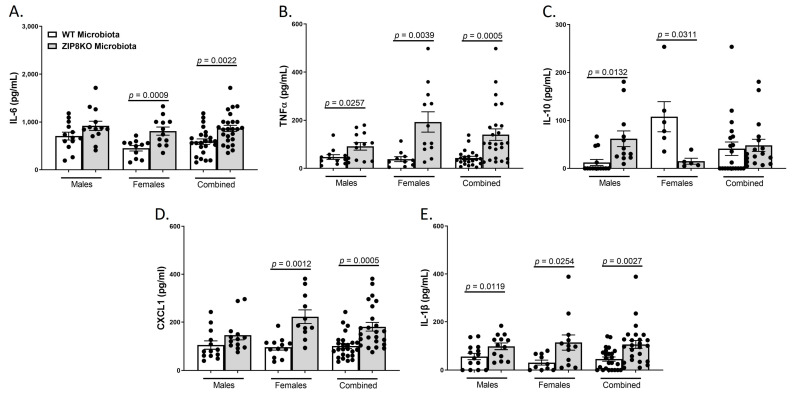
ZIP8-associated dysbiosis increases pulmonary inflammation. F1-microbiota mice were infected with *S. pneumoniae*, and the levels of BALF cytokines were assessed. (**A**) IL-6 levels from BALF. (**B**) TNFα levels from BALF. (**C**) IL-10 levels from BALF. (**D**) CXCL1 levels from BALF. (**E**) IL-1β levels from BALF. Bars represent the mean ± SEM, and dots represent individual mice. *p*-values are indicated in the figure and were determined by Welch’s *t*-test.

Consistent with leukocyte infiltration, pneumococcal infection was also associated with a significant increase in the expression of IL-6, TNF-α, CXCL1, IL-10, and IL-1β when these data were combined for both genders in both groups and was significantly higher in the F1 ZIP8KO-microbiota group when compared to F1 WT-microbiota animals (*p* ≤ 0.002; Figure 7A–E) There were gender specific differences but in all cases with similar trends. Taken together, these results demonstrate an imbalance in host defense leading to alteration in the cellular landscape due to an exaggerated immune response to *S. pneumoniae* as a consequence of ZIP8-mediated intestinal dysbiosis.

### 3.4. ZIP8-Associated Dysbiosis Alters Lung Tissue Integrity and Increases Bacterial Burden 

**Figure 8:**
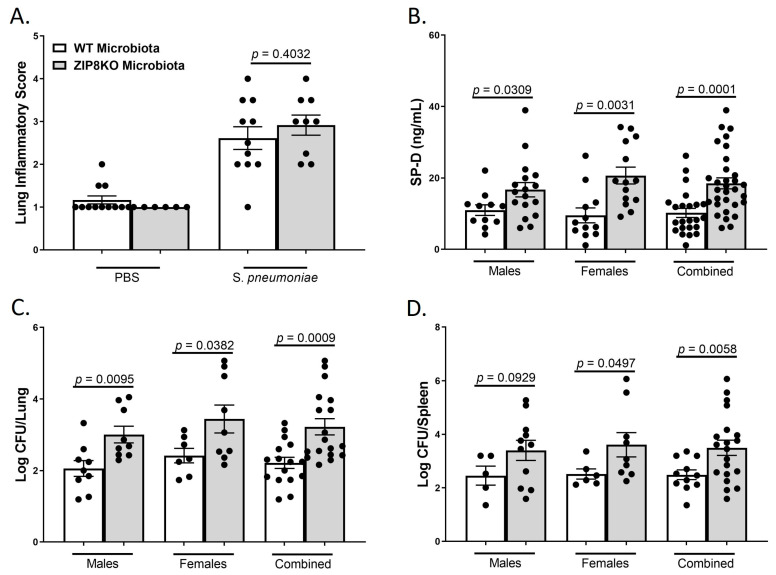
ZIP8-associated dysbiosis increases pulmonary damage, as well as pulmonary bacterial burden and dissemination. F1-microbiota mice were infected with *S. pneumoniae*, and pulmonary damage and bacterial burden were assessed. (**A**) Lung inflammatory scores via H&E histology. (**B**) Circulating levels of surfactant protein-D. Log transformation burden of *S. pneumoniae* in the (**C**) lungs and (**D**) spleen. Bars represent the mean ± SEM, and dots represent individual mice. *p*-values are indicated in the figure and were determined by Welch’s *t*-test.

To determine the role of ZIP8-mediated intestinal dysbiosis on pulmonary host defense against pneumococcal infection, both male and female F1 progeny (age 12–13 weeks) were administered 4 *×* 10^8^ CFU of *S. pneumoniae* in the lung and euthanized 48 h later to assess lung damage and bacterial burden. Histological staining of lung tissue revealed that both WT-microbiota and ZIP8KO-microbiota mice had increased lung injury post-pneumococcal infection, as determined by the corresponding lung inflammatory score (Figure 8A). However, F1 ZIP8KO-microbiota exhibited more pulmonary epithelial damage/permeability, as measured by circulating levels of surfactant protein-D (SP-D, a recognized biomarker of lung damage) (Figure 8B). To further determine whether increased lung tissue damage altered bacterial dissemination, bacterial counts were enumerated from the lungs and spleen of F1 mice. F1 ZIP8KO-microbiota mice exhibited a significantly higher bacterial burden in both the lung (Figure 8C) and spleen (Figure 8D) when compared to F1 WT-microbiota mice. These results are indicative of an increase in pulmonary permeability following *S. pneumoniae* infection, which could explain, in part, the increase in lung inflammation and cellular infiltration, as epithelial damage/permeability is known to increase inflammation and bacterial burden in the lungs and spleen.

### 3.5. ZIP8-Associated Dysbiosis Decreases Survival

**Figure 9:**
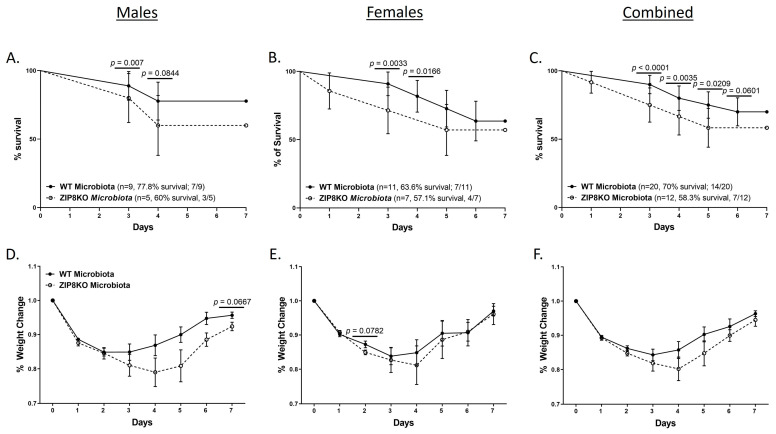
ZIP8-associated dysbiosis increases mortality following *S. pneumoniae* infection. F1-microbiota mice were infected with *S. pneumoniae*, and survival was assessed for 7 days post-infection. Survival post-infection in (**A**) males, (**B**) females, and (**C**) combined male and female mice. Percent weight change in (**D**) males, (**E**) females, and (**F**) combined male and female mice post-infection. Dots represent the mean and SD per group (n = 29 WT F1 mice, and n = 17 ZIP8KO F1 mice). *p*-values are indicated in the figure and were determined by the Chi-square test for each day post-infection.

**Figure 10:**
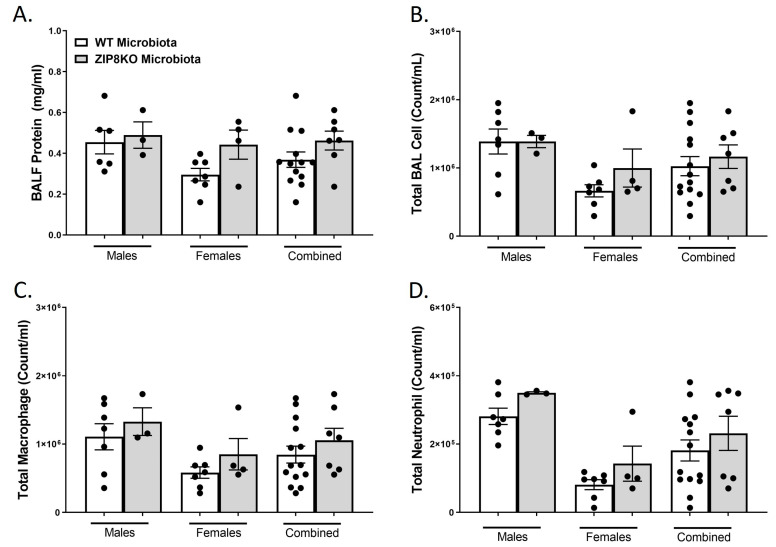
Increased pulmonary immune cells in ZIP8-associated dysbiosic mice persist for 7 days post-infection. F1-microbiota mice were infected with *S. pneumoniae*, and the number of BALF immune cells was assessed. (**A**) Protein levels from BALF. (**B**) Total BAL counts. (**C**) Total macrophages post-infection. (**D**) Total neutrophils post-infection. Bars represent the mean ± SEM, and dots represent individual mice.

To determine the role of ZIP8-mediated intestinal dysbiosis on survival after pneumococcal infection, germ-free mice were again colonized with the microbiota from WT and ZIP8KO mice. Male and female F1 progeny (age 12–13 weeks) were then administered 4 × 10^8^ CFU of *S. pneumoniae* in the lung via oropharyngeal aspiration, and survival was evaluated out to 7 days. Both F1 male and female ZIP8KO-microbiota mice had decreased survival in comparison to their WT counterparts (Figure 9A–C). In all groups, survivors demonstrated recovery, as shown by increased weight gain back to baseline between days 4 and 7. Specifically, all animals exhibited weight loss within the first 4 days post-infection (Figure 9D–F), with a trend toward more weight loss in F1 ZIP8KO-microbiota males followed by females in comparison to WT counterparts with both genders (Figure 9D–E). This was consistent with the appearance and activity levels with F1 ZIP8KO-microbiota males having the most significant symptoms. Enumeration of BAL total cell, neutrophil, macrophage counts, and protein levels at 7 days post-infection revealed no significant differences among all treatment groups regardless of gender (Figure 10A–D).

## 4. Discussion

Zinc is required for the growth and sustenance of eukaryotic and prokaryotic cells, thereby creating a “tug of war” for nutrient acquisition between the host and the microbe. Our group discovered that ZIP8 is indispensable in myeloid cell-mediated host defense against pneumococcus in the lung through the alteration of Zn-dependent cell-signaling networks that influence the immune response. Furthermore, it has been shown in humans and animal models that aging is a predisposing factor for Zn deficiency, which can adversely impact both innate and adaptive immunity. Further, we believe that our group was the first to investigate the impact of the Zn transporter-mediated regulation of the gut microbiome and its impact on pneumococcal pneumonia. Specifically, our laboratory demonstrated that ZIP8KO-associated intestinal dysbiosis was associated with both decreased levels of fecal butyrate and exacerbated bacterial pneumonia. However, given the limitations of antibiotic depletion prior to conventionalization (off-target effects of antibiotics and reproducibility between repeated conventionalizations), we have developed a novel translationally relevant ZIP8KO-microbiota mouse model, which allows us to systematically evaluate the immunological effects of the loss of Zip8 that are mediated by Zip8-associated microbiota changes independent of the direct effects of genetics. Specifically, we colonized six breeding pairs (three breeding pairs from three ZIP8KO cecal samples and three from WT cecal samples) of germ-free mice with the cecal microbiota from ZIP8KO mice or WT counterparts. Initial analysis found that colonized germ-free mice clustered differently as a result of two different microbiota cecal samples. In addition, microbial community structure was maintained in the F1 and F2 generation of colonized mice. This suggests that the compositional changes that are seen following the loss of ZIP8 in myeloid cells are transmissible across generations. Further, we found that both male and female F1 and F2 mice clustered based on the original donor microbiota samples. Finally, utilizing ZIP8KO-microbiota-associated F1 mice, we found that mice colonized with the cecal microbial communities from ZIP8KO mice had increased susceptibility to pneumococcal pneumonia when compared to mice colonized with cecal microbiota from WT mice, as determined by increased survival/mortality, pulmonary bacterial burden, bacterial dissemination, lung damage/leak, as well as increases in pulmonary immune cell infiltration and inflammation. Further, this new model system provided several key findings that supported and advanced our previous studies: (1) increased susceptibility to bacterial pneumonia due, in part, to the intestinal microbiota is an inheritable trait, as two generations of mice post-conventionalization had GI community structures similar to the founder genetic KO mice; (2) ZIP8KO mice, as well as ZIP8KO-associated microbiota mice had increased GI goblet cell numbers and altered goblet cell content; (3) ZIP8KO-associated dysbiosis contributed to lower levels of lung tissue Zn content; (4) ZIP8KO-associated microbiota increased susceptibility to respiratory infection in male mice; and finally (5) ZIP8KO-associated microbiota increased morbidity and mortality following *S. pneumoniae* infection. These new findings, coupled with our new model system, which eliminates confounding by off-target effects of antibiotics, provide a better framework for understanding the mechanisms by which ZIP8KO-associated dysbiosis increases susceptibility to respiratory infection.

The importance of the intestinal microbiota in optimal host defense against bacterial and viral respiratory infections is now well established [37,38,39,40,41,42,43,44]. In addition, multiple studies have utilized both germ-free mice and antibiotic-treated mice to evaluate susceptibility to pulmonary infections with bacterial pathogens, such as *K. pneumoniae* and *S. pneumoniae*. However, we believe that we are the first group to evaluate the longitudinal (inherited microbiota) effects of intestinal dysbiosis on pulmonary host defense. Specifically, the use of F1 microbiota-associated mice is quite novel and represents a new method to evaluate the effects of intestinal microbial communities on host health by accounting for the immune and physiological defects seen in germ-free mice. Several additional novel findings emerged from this study. First, ZIP8KO mice, as well as F1 ZIP8KO-microbiota (although not significant) mice, exhibited marked increases in the number and percentage of colonic goblet cells and a specific loss of the goblet cells with acidic mucins. Second, F1 ZIP8KO-microbiota mice exhibited marked decreases in pulmonary Zn levels. These findings were surprising and suggested that the myeloid-specific loss of ZIP8 and the associated changes in the intestinal microbial communities influence both tissue-specific Zn levels and changes in intestinal mucin production. Coupled with our previous data that demonstrate that ZIP8KO-associated intestinal dysbiosis was associated with a significant decrease in the levels of cecal butyrate, this suggests a dynamic and synergistic role of ZIP8 and the intestinal microbiota in maintaining GI health and systemic metal uptake. For example, goblet cells not only produce mucus but also are intimately linked to the immune system and are synchronized with microbial colonization. Altered goblet cell number and/or function have been associated with both infectious and inflammatory conditions. These data suggest that the GI associated changes in goblet cells likely play a role in altering the composition of the GI microbiota and likely provide an additional inflammatory insult, which together increases susceptibility to infection. In addition, these data also suggest that the microbiota changes associated with altered goblet cell number do not significantly feedback or contribute to goblet cell hyperplasia. However, several critical questions remain to be investigated. What specific cell types and associated signaling pathways are adversely impacted by alterations of butyrate and likely other metabolites? What is the role of altered GI mucin production on pulmonary host defense, as well as its effects on butyrate production? Finally, how do alterations in the GI microbial communities contribute to changes in Zn levels in distal organs such as the lung? Finally, given the high incidence of both nutritional Zn deficiency and the ZIP8 variant dysfunctional allele (rs13107325; Ala391Thr risk allele), can Zn supplementation or bacterial metabolite reconstitution, with butyrate or other analogs, be used to bolster host defense in at-risk populations, thereby reducing the high morbidity and mortality associated with pneumococcal pneumonia?

## 5. Conclusions

Disease-associated intestinal microbial communities can be inherited across multiple generations and contribute to impaired host immune responses. Specifically, we demonstrated that F1 ZIP8KO-microbiota-associated mice had increased susceptibility to *Streptococcus* pneumonia when compared to mice colonized with cecal microbiota from WT mice, as determined by increased survival/mortality, pulmonary bacterial burden, bacterial dissemination, lung damage/leak, as well as increases in pulmonary immune cell infiltration and inflammation. Future studies will seek to understand the mechanisms by which the intestinal microbiota influences host defense, with a specific focus on understanding the crosstalk between myeloid cells, intestinal goblet cells, the microbiota, SCFAs, and tissue-specific Zn levels, during bacterial pneumonia. This, in turn, we envision, will lead to new screening methods to identify at-risk populations and subsequent innovative strategies to reduce the burden of CAP.

## Data Availability

Sequencing data have been deposited in the National Center for Biotechnology Information Sequence Read Archive (BioProject ID: PRJNA942295). All additional data which support the findings of this study are available within the paper.

## Supplementary Materials

The following supporting information can be downloaded at: https://www.mdpi.com/article/10.3390/pathogens12050639/s1, Figure S1: Myeloid-specific loss of Zip8 shows no differences in colon histology; Figure S2: Zip8-associated dysbiosis does not alter the colon histology.
