# Supplementary figures and images for: The Inherited Intestinal Microbiota from Myeloid-Specific ZIP8KO Mice Impairs Pulmonary Host Defense against Pneumococcal Pneumonia
Source: Pathogens. 2023 Apr 25;12(5):639. doi: 10.3390/pathogens12050639 (PMC10222741; doi:10.3390/pathogens12050639)

A.

WT

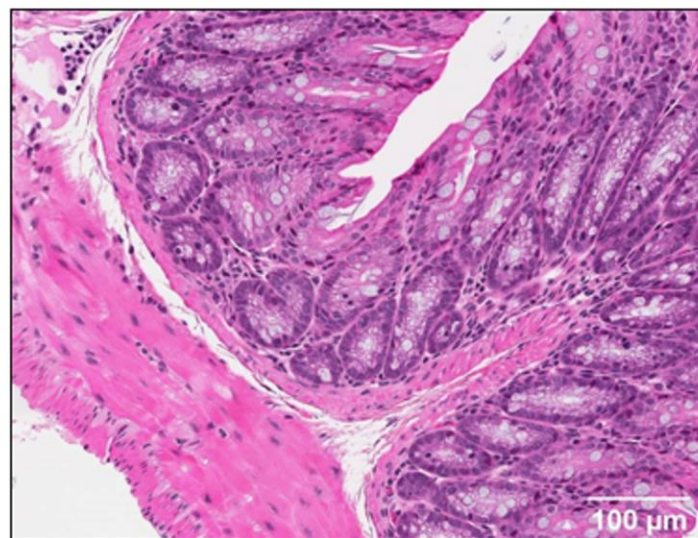

ZIP8KO

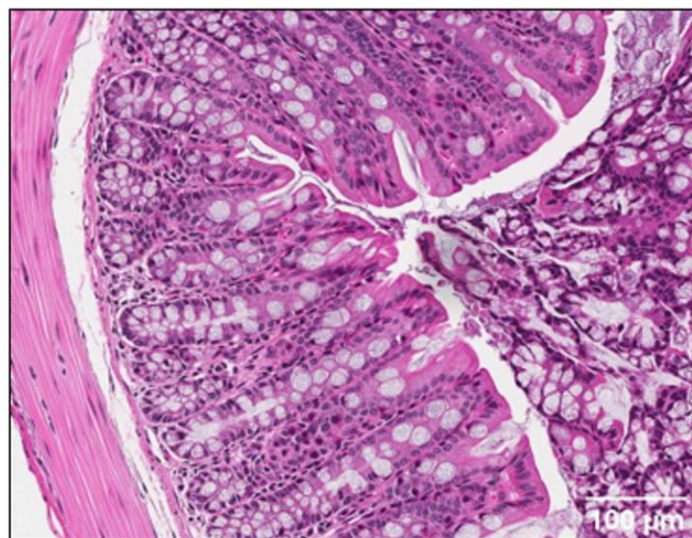

B.

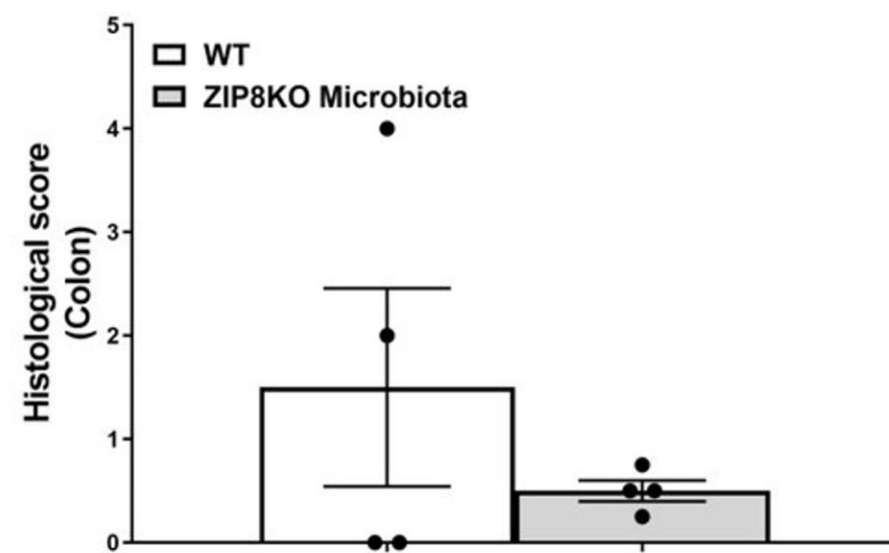

Supplement: Supplementary file 1 [file pathogens-12-00639-s001.zip › Figure S1.pdf]

A.

F1 WT Microbiota

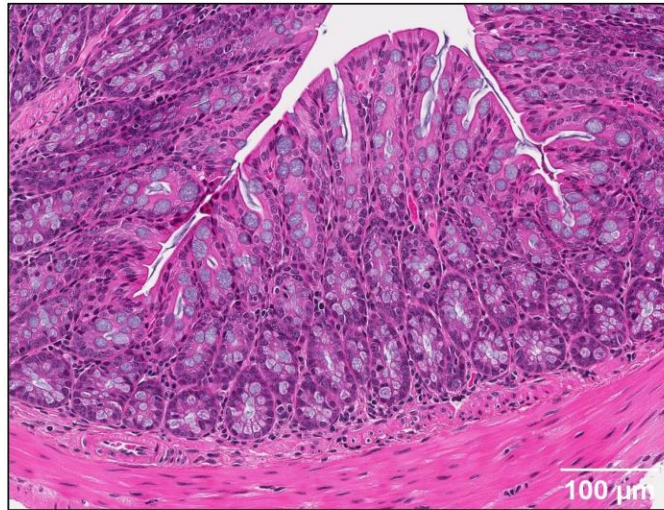

F1 ZIP8KO Microbiota

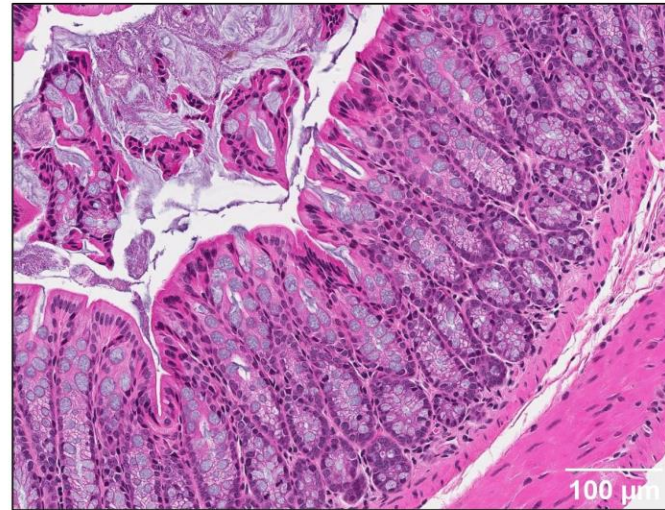

B.

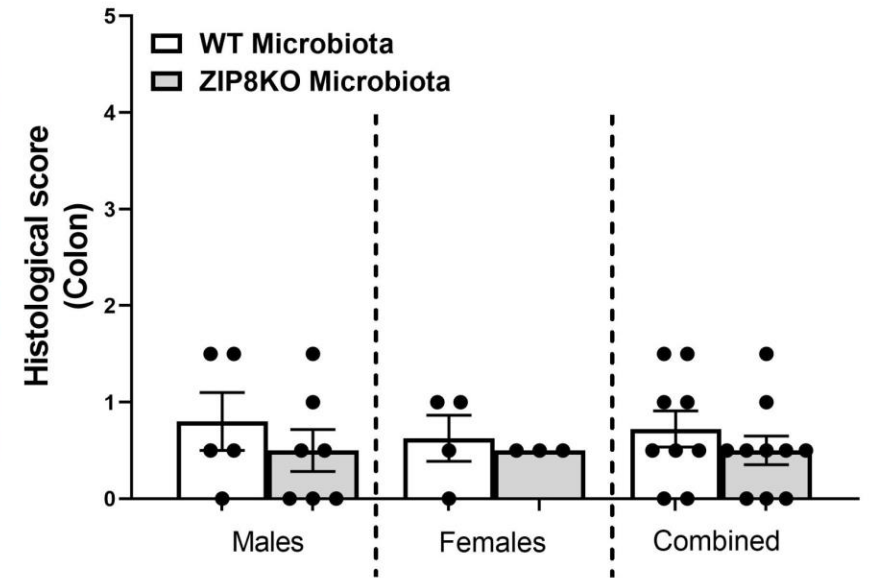

Supplement: Supplementary file 1 [file pathogens-12-00639-s001.zip › Figure S2.pdf]
